# Community management: An effective model to reduce medication discontinuation rate in patients with schizophrenia

**DOI:** 10.1371/journal.pone.0324114

**Published:** 2026-01-09

**Authors:** Hua Ren, Qin Yang, Changjiu He, Jian Jiao, Zaiquan Dong

**Affiliations:** 1 The Clinical Hospital of Chengdu Brain Science Institute, MOE Key Lab for Neuroinformation, School of Life Science and Technology, University of Electronic Science and Technology of China, Chengdu, P.R. China; 2 Sleep Medicine Center, Mental Health Center, Translational Neuroscience Center, West China Hospital, Sichuan University, Chengdu, P.R. China; 3 Mental Health Center, National Center for Mental Disorders, West China Hospital, Sichuan University, Chengdu, P.R. China; Federal University of Paraiba, BRAZIL

## Abstract

**Background:**

Medication discontinuation is highly prevalent among patients with schizophrenia and is associated with poor clinical outcomes. Although community-based management models have been implemented in China to support patients, recent evidence of their effectiveness in reducing medication discontinuation rates remains limited.

**Methods:**

In this cross-sectional study, we surveyed 1,531 patients with schizophrenia under community management in Chengdu, China, using a multi-stage sampling approach. Data on treatment discontinuation (defined as cessation of all antipsychotics for >15 days without medical advice), socio-demographics, clinical characteristics, and service utilization were collected via face-to-face interviews. Univariate and multivariable logistic regression analyses were used to identify the factors associated with medication discontinuation.

**Results:**

The overall rate of medication discontinuation was 4.1%. Multivariable analysis identified several independent risk factors for discontinuation: weak stable disease state (adjusted odds ratio [aOR] = 2.70, 95% confidence interval [CI]: 1.34–5.24), lack of insight (aOR for partial vs. no insight = 0.25, 95% CI: 0.08–0.80; aOR for full vs. no insight = 0.18, 95% CI: 0.05–0.65), presence of noticeable side effects (aOR = 1.98, 95% CI: 1.05–3.64), and absence of regular follow-up (aOR for intermittent vs. no follow-up = 0.38, 95% CI: 0.20–0.72; aOR for regular vs. no follow-up = 0.22, 95% CI: 0.10–0.44).

**Conclusion:**

Community management was associated with a low rate of medication discontinuation in patients with schizophrenia. Key modifiable factors, including disease stability, insight, medication side effects, and follow-up adherence should be prioritized in community-based interventions to further improve treatment continuity.

## 1 Introduction

Schizophrenia is a severe psychiatric disorder characterized by cognitive deficits, disordered thinking, emotional abnormalities, and multiple functional defects with a high recurrence rate and significant harmful effects [1]. A 2019 cross-sectional epidemiological study of a nationally representative adult sample across 157 surveillance sites in 31 provinces revealed that the lifetime and 12-month prevalence of schizophrenia in China were 0.6% [2]. The disability-adjusted life years (DALYs) for schizophrenia in China are estimated at approximately 3.45 million, with an age-standardized DALY rate of 203.88 per 100,000 people, which is the highest globally [3,4]. Further analyses highlight the profound personal and economic impacts of the disease. A 2020 burden-of-disease analysis reported that compared to the age- and sex-matched general population, Chinese individuals with schizophrenia had a reduced life expectancy of 20.6 years, lost 18.4 QALYs, and incurred average direct medical costs approximately three times higher [5]. This burden extends substantially to families. Yu et al. reported that over half (52%) of caregivers reported a moderate-to-severe family burden, predominantly financial [6]. This is exacerbated by productivity losses, as 28% of the patients are unable to work. An analysis in rural China quantified this, showing that while the average annual household income was ¥12,108, the disease imposed an average annual direct and indirect financial burden of ¥963 and ¥11,724, respectively [7,8].

Antipsychotics are essential for schizophrenia treatment; however, patient adherence is often low. Interrupting antipsychotic medications, even for a short time, reduces treatment effectiveness [9–11], increases the risk of relapse [12–15], and increases psychiatric hospital admissions [16,17]. Although continued medication is important, only about half of patients with schizophrenia adhere to antipsychotic medication. Medication nonadherence in schizophrenia is influenced by a confluence of factors across several domains: patient-related factors (e.g., younger age, poor insight, cognitive impairments, low education, low socioeconomic status, and substance abuse); illness-related factors (e.g., high intensity of delusional symptoms); treatment-related factors (e.g., negative medication attitudes, poor therapeutic alliance, and irregular attendance to appointments); and psychosocial factors (e.g., internalized stigma, disbelief in recovery, and experience of barriers to care) [18,19].

Effective medication management can reduce discontinuation risk [17], especially in community-based models that improve adherence and reduce violent behavior [20–23]. Community-based service models help patients maintain their connections with healthcare institutions and increase their access to community services [24]. The main process of this working model is to ensure the continuity of patients’ treatment through continuous assessment and satisfaction of patients’ medical needs, so as to facilitate control of the disease, improve social function, reduce hospitalization, shorten hospitalization time, and improve the quality of life of caregivers and patients [25]. Current models of community mental health services include community mental health care centers, day clinics, self-help communities, and enhanced case management [26]*.*

A case management model is commonly used in community-based services with the ultimate goal of providing comprehensive mental health services to achieve individual goals and provide the most appropriate social functioning and good quality of life in line with patients’ individual needs [27]. This model has been shown to significantly promote clinical recovery, improve social and occupational functioning, and reduce the length of hospital stay of patients with schizophrenia [24].

Since 2004, the People’s Republic of China has implemented measures to manage severe mental disorders, including schizophrenia [23]. In 2009, the management of six major mental disorders including schizophrenia, was integrated into the National Public Health service system [28]. This is mainly based on the Assertive Community Treatment in America and Japan [29] with localized improvements. By 2025, > 80% of counties are expected to offer extensive community rehabilitation services for mental disorders with the aim of establishing a continuous prevention and treatment model primarily based on community management. Local health institutions provide services under this model, including health record management, follow-up, case management, medication guidance, and emergency medical assistance, with a focus on patients with schizophrenia. Although community-based services have been carried out in China for many years and have proven to be helpful for severe mental disorders, including schizophrenia [17], there is a lack of new evaluations on the implementation effect of community-based services in recent years, and especially the impact of reducing the medication discontinuation rate of patients has not been reported.

In this study, we investigated the medication status of patients with schizophrenia under community management and analyzed related factors to verify that community-based management is an effective model for mental illness management that is well suited for reducing medication interruptions. The study’s findings not only offer community health workers practical strategies for prioritizing high-risk patients and optimizing follow-up care to improve individual outcomes but also provide policymakers with robust evidence supporting the model’s nationwide expansion, thereby potentially reducing the overall societal and economic burden of schizophrenia.

## 2 Participants and methods

### 2.1 Participants

The study surveyed registered patients with schizophrenia who were managed by community administrators and met the diagnostic criteria for schizophrenia outlined in the International Statistical Classification of Diseases and Related Health Problems, 10th Revision. This study was approved by the Ethics Committee on Biomedical Research of West China Hospital (approval number: 2020−797) and conducted in accordance with the Declaration of Helsinki. Probability proportional to size was used to select the sampling areas (one urban district and one suburban district in Chengdu, China). All the schizophrenia patients registered in the Community Management program within these two districts were included in this survey. The survey was conducted from September 1–30, 2020, and 1,531 valid questionnaires were collected. Before enrollment, formal verbal consent procedures were systematically implemented and audio-recorded confirmation was obtained from each participant. The informed consent process was conducted by specially trained research team members with expertise in communicating with individuals with mental disorders and their families. The process involved both patients and their legal guardians. Informed consent was obtained from the patient whenever they had partial or full decision-making capacity, and independent written informed consent was obtained from their legal guardian. The recruitment process including:

(1) Identification of eligible patients from community health registries.
(2) Initial contact via community health workers.
(3) Joint consent process involving patients and guardians.
(4) Face-to-face interviews conducted by trained investigators.

### 2.2 Methodology

Based on previous studies [28,30], the questionnaire was designed by experienced psychiatrists and included age, sex, education, residence, income, reimbursement ratio for drug insurance, employment status, disease course, disease state, insight, drug knowledge, drug type, side effects, outpatient follow-up at the hospital, and family support.

- Medication discontinuation: The normative definition of medication discontinuation is difficult [31]; in this study, it was defined as failure to take any antipsychotic medication for more than 15 days within the past month without medical advice or due to external factors, such as the coronavirus disease 2019 pandemic [32]. A 15-day threshold was chosen based on clinical guidelines and prior studies suggesting that even short interruptions beyond two weeks significantly increase relapse risk and disrupt therapeutic steady-state [33]. Patients treated with long-acting injectable antipsychotics were excluded. Patients were classified into the discontinuation and non-discontinuation groups based on whether the medication was interrupted.
- Family income evaluation: This referred to the total monthly income of major family members.
- Employment status: Individuals who were able to work for most of the preceding year and had fixed income were defined as employed; otherwise, they were considered unemployed.
- Disease state: This was divided into stable, weakly stable, and unstable states according to the fluctuation of symptoms. A “stable” status was defined as one in which psychiatric symptoms had largely resolved, insight was largely restored, social functioning was at a fair or good level, with no serious adverse drug reactions and no risk of harmful or disruptive behavior. A “weak stable” status described patients who were essentially stable but still exhibited impairments in at least one of the following domains: psychiatric symptoms, insight, or social functioning. An “unstable” status was defined as the presence of overt psychiatric symptoms, lack of insight, serious adverse drug reactions, or an identifiable risk of harmful or disruptive behavior. For all participants who met the criteria for medication discontinuation, their pre-discontinuation clinical status was confirmed by their guardians.
- Insight: This was based on the cognitive understanding of the patients’ psychological state and was categorized as none, partial, or full insight.
- Knowledge about drugs: This was divided into three categories (good, average, and poor) according to the patient’s understanding of the importance of drug maintenance therapy, effect of drug treatment, common side effects, and precautions for administration.
- Side effects: This variable was based on the main complaints of patients and divided into two categories (yes or no).
- Drug type: This refers to the number and types of medications taken by patients concurrently.
- Follow-up: Regular follow-up included patients who visited their physicians according to their medical or self-arranged schedules. Intermittent follow-up referred to patients whose visits were irregular and were based on illness changes rather than prearranged appointments. No follow-up indicated that there was neither a physician appointment nor a self-arranged visit.
- Family support: This refers to the totality of ongoing material and psychosocial resources that patients receive from their core family members (such as spouses, parents, or children) to aid in illness management and recovery.

### 2.3 Quality control

The investigators were trained to ensure consistency and reliability in the data collection and verification. Each investigator explained the purpose of the study to the participants in the presence of the main caregiver and conducted the face-to-face surveys. The questionnaire was collected onsite to ensure the authenticity and reliability of the data. Data entry was performed by one investigator and independently verified by another, and any inconsistencies were resolved through review by a third investigator.

### 2.4 Statistical analysis

All statistical analyses were performed using SPSS version 26.0. The chi-square test or Fisher’s exact test was used to identify potential predictive and associated factors. The statistical significance of the differences between the two groups was determined using either the chi-square test or Fisher’s exact test, as appropriate. Logistic regression analyses were performed using three sequential models. Covariate selection based on prior literature and clinical relevance and the assessment of multicollinearity using variance inflation factors (VIF < 2.0 for all variables). In Model 1, sex and age were entered into the equation. In Model 2, education, residence, monthly household income, payments, and employment were added. In Model 3, disease course, disease state, insight level, drug knowledge, side effects, family support, medication type, and follow-up were included. All variables were controlled for at each step of the logistic regression analysis.

## 3 Results

### 3.1 Descriptive data of the included participants

In total, 1,609 individuals were included in this study. After excluding 78 participants who declined to participate and two who were ineligible due to insufficient data, a final sample of 1,531 patients with schizophrenia was included in the analysis. The age at assessment ranged from 18 to 65 years in the medication discontinuation group (n = 63) and from 18 to 77 years in the non-discontinuation group (n = 1468). Significant differences were observed between the two groups in disease state, type of medication (monotherapy vs. combination therapy), side effects, drug knowledge, insight, and follow-up status (all *P* < 0.05; Table 1).

**Table 1:** Demographic and clinical characteristics between the non-discontinuation group and the discontinuation group.

	All	Non-discontinuation group	Discontinuation group	p value
Number of participants	1531	1468	63	
Sex				0.737
Female	894 (58.4%)	859 (58.5%)	35 (55.6%)	
Male	637 (41.6%)	609 (41.5%)	28 (44.4%)	
Age (years)				0.669
0-29	211 (13.8%)	203 (13.8%)	8 (12.7%)	
30-39	328 (21.4%)	315 (21.5%)	13 (20.6%)	
40-49	424 (27.7%)	409 (27.9%)	15 (23.8%)	
50-59	310 (20.2%)	298 (20.3%)	12 (19.0%)	
≥ 60	258 (16.9%)	243 (16.6%)	15 (23.8%)	
Education				0.647
< Senior high school	1132 (73.9%)	1086 (74.0%)	46 (73.0%)	
Senior high school	230 (15.0%)	222 (15.1%)	8 (12.7%)	
College or undergraduate	169 (11.0%)	160 (10.9%)	9 (14.3%)	
Residence				0.453
Urban areas	481 (31.4%)	458 (31.2%)	23 (36.5%)	
Rural areas	1050 (68.6%)	1010 (68.8%)	40 (63.5%)	
Monthly household income				0.101
< 5000	1141 (74.5%)	1088 (74.1%)	53 (84.1%)	
≥ 5000	390 (25.5%)	380 (25.9%)	10 (15.9%)	
Payment				0.061
Self-pay	150 (9.8%)	139 (9.5%)	11 (17.5%)	
Medical insurance	1381 (90.2%)	1329 (90.5%)	52 (82.5%)	
Employment				0.119
No	1176 (76.8%)	1122 (76.4%)	54 (85.7%)	
Yes	355 (23.2%)	346 (23.6%)	9 (14.3%)	
Family support				0.058
Poor	80 (5.2%)	75 (5.1%)	5 (7.9%)	
General	562 (36.7%)	532 (36.2%)	30 (47.6%)	
Good	889 (58.1%)	861 (58.7%)	28 (44.4%)	
Disease course (years)				0.446
0-5	406 (26.5%)	390 (26.6%)	16 (25.4%)	
5-10	482 (31.5%)	466 (31.7%)	16 (25.4%)	
> 10	643 (42.0%)	612 (41.7%)	31 (49.2%)	
Disease state				<0.001
Stable	1357 (88.6%)	1315 (89.6%)	42 (66.7%)	
Weak stable	159 (10.4%)	140 (9.5%)	19 (30.2%)	
Unstable	15 (1.0%)	13 (0.9%)	2 (3.2%)	
Medication type				0.011
Single	429 (28.0%)	402 (27.4%)	27 (42.9%)	
Combined	1102 (72.0%)	1066 (72.6%)	36 (57.1%)	
Side effects				<0.001
No	1289 (84.2%)	1249 (85.1%)	40 (63.5%)	
Yes	242 (15.8%)	219 (14.9%)	23 (36.5%)	
Drug knowledge				<0.001
Poor	159 (10.4%)	142 (9.7%)	17 (27.0%)	
General	930 (60.7%)	894 (60.9%)	36 (57.1%)	
Good	442 (28.9%)	432 (29.4%)	10 (15.9%)	
Insight level				<0.001
No	27 (1.8%)	19 (1.3%)	8 (12.7%)	
Partial	539 (35.2%)	510 (34.7%)	29 (46.0%)	
Full	965 (63.0%)	939 (64.0%)	26 (41.3%)	
Follow up				<0.001
No	250 (16.3%)	221 (15.1%)	29 (46.0%)	
Intermittent	658 (43.0%)	637 (43.4%)	21 (33.3%)	
Regular	623 (40.7%)	610 (41.6%)	13 (20.6%)	

Note: Values are n (%).

### 3.2 Factors associated with medication discontinuation

Univariate and multivariable regression analyses were performed using medication discontinuation as the dependent variable (non-discontinuation = 0; discontinuation = 1) and age, sex, education, residence, income, payment methods, employment, disease course, disease state, insight, drug knowledge, medication types, side effects, follow-up, and family support as independent variables. The results showed that an unstable or weakly stable disease state, lack of insight, noticeable medication side effects, and absence of regular follow-up were significant risk factors for medication discontinuation (all *P *< 0.05; Table 2).

**Table 2:** Adjusted odds ratios (aOR) for medication discontinuation (discontinuation = 1).

	Reference (1.00)	Category (these categories vs. reference)	Model 1^a^		Model 1^b^		Model 1^c^	
			aOR (95% CI)	p value	aOR (95% CI)	p value	aOR (95% CI)	p value
Intercept			0.04 (0.02–0.07)	<0.001	0.11 (0.03–0.33)	<0.001	1.34 (0.20–8.47)	0.761
Sex	Female	Male	1.15 (0.68–1.90)	0.600	1.01 (0.56–1.79)	0.973	1.31 (0.69–2.45)	0.403
Age (years)	0-29	30-39	1.04 (0.43–2.67)	0.931	1.13 (0.45–2.97)	0.798	0.73 (0.27–2.07)	0.543
	0-29	40-49	0.92 (0.39–2.32)	0.856	1.12 (0.45–2.96)	0.812	0.71 (0.25–2.11)	0.524
	0-29	50-59	1.02 (0.41–2.64)	0.973	1.24 (0.46–3.52)	0.671	0.65 (0.21–2.11)	0.465
	0-29	≥60	1.57 (0.67–3.96)	0.317	2.07 (0.76–5.98)	0.163	0.93 (0.28–3.17)	0.905
Education	<Senior high school	Senior high school			1.04 (0.43–2.30)	0.918	1.09 (0.42–2.56)	0.848
	<Senior high school	College or undergraduate			1.78 (0.73–4.03)	0.182	1.62 (0.63–3.90)	0.299
Residence	Urban areas	Rural areas			0.82 (0.47–1.49)	0.508	0.91 (0.49–1.76)	0.782
Monthly household income	<5000	≥5000			0.51 (0.24–0.99)	0.063	0.59 (0.26–1.18)	0.158
Payment	Self-pay	Medical insurance			0.49 (0.26–1.03)	0.044	0.71 (0.34–1.60)	0.380
Employment	No	Yes			0.59 (0.26–1.19)	0.167	0.64 (0.27–1.40)	0.291
Disease course (years)	0-5	5–10					0.90 (0.41–1.95)	0.781
	0-5	>10					0.91 (0.43–2.00)	0.815
Disease state	Stable	Weak stable					2.70 (1.34–5.24)	0.004
	Stable	Unstable					1.55 (0.20–7.49)	0.621
Insight level	No	Partial					0.25 (0.08–0.80)	0.016
	No	Full					0.18 (0.05–0.65)	0.007
Drug knowledge	Poor	General					0.87 (0.41–1.94)	0.731
	Poor	Good					0.72 (0.26–2.00)	0.528
Side effects	No	Yes					1.98 (1.05–3.64)	0.030
Family support	Poor	General					0.92 (0.31–3.26)	0.891
	Poor	Good					0.71 (0.25–2.50)	0.559
Medication type	Single	Combined					0.64 (0.36–1.15)	0.130
Follow up	No	Intermittent					0.38 (0.20–0.72)	0.003
	No	Regular					0.22 (0.10–0.44)	<0.001

Note:

^a^Model 1 with adjustment for sex and age.

^b^Model 2 with adjustment for covariates in model 1 plus education, residence, monthly household income, payment, and employment.

^c^Model 3 with adjustment for covariates in model 2 plus disease course, disease state, insight level, drug knowledge, side effects, family support, medication type, and follow up.

Abbreviations: CI = confidence interval.

## 4 Discussion

The need for supervised drug use management in patients with schizophrenia remains unclear. Leaving medication solely to patient discretion or guardian supervision would be disadvantageous given the mixed public perception of the disease. A community-based management model may be an effective method to reduce the probability of medication interruption in patients with schizophrenia.

The current study, conducted in Chengdu, showed that the medication discontinuation rate of patients with schizophrenia under community management was 4.1%. Given the lack of consistent and agreed-upon definitions of this phenomenon, it is difficult to compare these results with those of similar studies. In a prospective, multicenter, open-cohort, real-world study, treatment discontinuation was defined as a month of poor medication adherence. The study reported that over 18 months, the treatment discontinuation rate was 80.4% for monotherapy and 81.1% for polypharmacy [34]. Another study applied the criterion that defined medication discontinuation as either a switch in medication or a gap of 60 days in the days of supply during the entire follow-up period. Within the 1-year follow-up, 72% of patients with schizophrenia using oral antipsychotics experienced treatment discontinuation, according to this definition [35]. Mojtabai et al. reported that 65% of patients discontinued treatment over 48 months, defining discontinuation as a period of ≥2 weeks without antipsychotic medication [33]. A randomized controlled trial reported that 55.2% of patients with schizophrenia receiving conventional therapy discontinued treatment within 180 days, defining discontinuation as a gap of >30 days in the prescription of the index drug [36]. Another study with 4 years of follow-up in the United States of America showed that 36% of patients had poor adherence to medication each year, and 61% discontinued their medication at some point during the follow-up period [37]. Based on current reports, the average adherence rate of patients with schizophrenia is approximately 40–60%, and some studies have reported that 75–90% of patients become nonadherent within 1–2 years after discharge. Valenstein et al. reported that effective management measures can significantly reduce the probability of medication interruption [38]. According to a study by İncedere et al., the implementation of a 24-month Case Management program was associated with an increase in patients’ voluntary medication adherence rates from 6.7% to 56.7% [24]. This suggests that factors at play may include supervision of medication, facilitation of access to medication, more opportunities for follow-up visits, and humanistic care from community managers.

The results of the multivariable logistic regression analysis showed that the discontinuation rates of medication during the weakly stable phase were 2.70 times higher than those in the stable disease stage (95% confidence interval: 1.34–5.24). Patients in the unstable phase are controlled by psychotic symptoms, such as hallucinations and delusions, are unable to properly assess their mental state, and refuse to take medication [39–41]. Thus, a stable disease state is necessary to reduce medication interruption. One study showed that patients with schizophrenia had high medication adherence when their disease was in the normal-to-mild stages (*P* = 0.015) [42]. The severity of symptoms and ability to recognize psychotic symptoms are independent risk factors for medication adherence [43].

Second, poor insight into the disease is a risk factor for medication discontinuation, which is consistent with the conclusions of most studies [44–46]. A good insight implies a proper understanding of the disease state and its adverse consequences, which is a crucial marker of disease recovery and an important condition for medication adherence. Consequently, improving patients’ insight into the disease is a major goal of schizophrenia treatment.

Furthermore, prominent side effects are an important cause of medication interruption, as indicated in previous studies [14,34,44,47]. This study did not investigate the types of medications taken by patients, such as first- and second-generation antipsychotics. Research has shown that second-generation antipsychotics, which often have milder side effects, may enhance medication compliance and reduce discontinuation rates [14,48]. Regular follow-up was another one of the factors associated with medication discontinuation. Although there is limited research on this aspect, it could be beneficial to adjust medications in a timely manner through regular follow-ups. Information about the disease, health education, and emotional support provided during follow-up visits may help patients better adhere to their medication regimens.

Additionally, a study has demonstrated that patients who receive emotional support from family members are more likely to adhere to psychopharmacological treatment than those who do not receive such support [49]. Proactive support from primary caregivers (e.g., parents) is crucial for positively influencing patients’ acceptance of their condition and overall quality of life [50]. However, the current study did not reach the same conclusion, suggesting that community management partially play a role as a support system for patients. Moreover, cognitive-behavioral therapy and other socio-psychological interventions can reduce the risk of rehospitalization and are effective in enhancing adherence and reducing medication discontinuation [14].

In summary, despite challenges such as lack of professional talent and low cooperation from patients or their families in community mental health management, the rate of medication discontinuation among patients with schizophrenia under community management is significantly lower than that in the general population. The risk factors leading to discontinuation are a weak stable disease status, lack of insight, severe side effects of drugs, and lack of regular follow-up, highlighting the key areas of community management for patients with schizophrenia. Our findings have significant implications for both practice and policy. For community care, they provide a practical tool to help workers prioritize high-risk patients and allocate resources efficiently. For public health, the model’s success in maintaining treatment supports its expansion across China, which would reduce the substantial economic and social burden of schizophrenia by preventing relapses.

This study has certain limitations. First, it did not consider differences in the types of medication, as studies have shown variations in discontinuation rates between first- and second-generation antipsychotics [51]. Second, it did not account for the effectiveness of the medication, particularly the subjective effects experienced by the patients, which requires further clarification in future research. Third, objective assessments of medication discontinuation, such as blood or urine medication levels, were not performed, which may have caused a bias. Fourth, the measures used in this study were largely dependent on subjective evaluations from the participants or their guardians, which could be a source of bias. The cross-sectional design of this study did not allow the assessment of patients’ medication adherence behaviors over time. Although this methodological approach is useful for examining associations between variables, causality cannot be established. Furthermore, this study was conducted in two districts of Chengdu and excluded patients on long-acting injectable antipsychotics, which may limit the generalizability of our findings to other regions or treatment modalities.

## Supporting information

S1 File (XLSX)
